# Culture of iPSCs Derived Pancreatic *β*-Like Cells In Vitro Using Decellularized Pancreatic Scaffolds: A Preliminary Trial

**DOI:** 10.1155/2017/4276928

**Published:** 2017-04-05

**Authors:** Jian Wan, Yan Huang, Pengcheng Zhou, Yibing Guo, Cen Wu, Shajun Zhu, Yao Wang, Lei Wang, Yuhua Lu, Zhiwei Wang

**Affiliations:** ^1^Department of General Surgery, Affiliated Hospital of Nantong University, Nan Tong, Jiang Su, China; ^2^Research Center of Clinical Medicine, Affiliated Hospital of Nantong University, Nan Tong, Jiang Su, China; ^3^Department of General Surgery, Affiliated Cancer Hospital of Nantong University, Nan Tong, Jiang Su, China; ^4^Department of Emergency Surgery, Affiliated Hospital of Nantong University, Nan Tong, Jiang Su, China

## Abstract

Diabetes mellitus is a disease which has affected 415 million patients in 2015. In an effort to replace the significant demands on transplantation and morbidity associated with transplantation, the production of *β*-like cells differentiated from induced pluripotent stem cells (iPSCs) was evaluated. This approach is associated with promising decellularized scaffolds with natural extracellular matrix (ECM) and ideal cubic environment that will promote cell growth in vivo. Our efforts focused on combining decellularized rat pancreatic scaffolds with mouse GFP^+^-iPSCs-derived pancreatic *β*-like cells, to evaluate whether decellularized scaffolds could facilitate the growth and function of *β*-like cells. *β*-like cells were differentiated from GFP^+^-iPSCs and evaluated via cultivating in the dynamic circulation perfusion device. Our results demonstrated that decellularized pancreatic scaffolds display favorable biochemical properties. Furthermore, not only could the scaffolds support the survival of *β*-like cells, but they also accelerated the expression of the insulin as compared to plate-based cell culture. In conclusion, these results suggest that decellularized pancreatic scaffolds could provide a suitable platform for cellular activities of *β*-like cells including survival and insulin secretion. This study provides preliminary support for regenerating insulin-secreting organs from the decellularized scaffolds combined with iPSCs derived *β*-like cells as a potential clinical application.

## 1. Introduction

According to the international diabetes foundation, by 2015, 415 million adults have suffered from diabetes worldwide, with this number expected to rise to 642 million by 2040. Type 1 diabetes mellitus (T1DM) is characterized by an absolute lack of insulin and thus is dependent on exogenous insulin treatment. However, periodic exogenous insulin injections cannot accurately regulate blood glucose levels and, despite treatment, those affected continue to suffer ill effects [1, 2] such as diabetic nephropathy, neuropathy, retinopathy, and arteriosclerosis. As such, the best current practice for treating diabetes involves transplantation of the islet or pancreas [3–5], but the large numbers of patients, limited supply of donor tissue, the risk and cost of operation, and necessity for lifelong immunosuppression have limited the application of this treatment modality. *β*-like cells differentiated from embryonic stem cells (ES) [6], induced pluripotent stem cells (iPSCs) [7], and mesenchymal stem cells (MSC) [8] have become the most promising solution by providing a potentially inexhaustible means of generating *β* cells for transplantation and with low immunogenicity. Recently, Alipio et al. successfully induced iPSCs to differentiate into *β*-like cells [9]. Most notably, their results demonstrated amelioration of the hyperglycemic phenotype within the mouse models.

However, recent studies have shown that implanted *β*-like cells cannot adapt to the environment and can be easily removed by instant blood-mediated inflammatory reaction (IBMIR) [10]. While some biomaterial scaffolds have been explored to promote *β*-like cells engraftment and survival, none of them can factually simulate the natural growth in vivo due to the lack of vasculature for delivery of oxygen, nutrients, and removal of metabolite. As such, it remains essential to identify ideal extracellular microenvironment for *β*-like cells to survive and work effectively. Thanks to the cutting-edge technology of three-dimensional decellularized bioscaffold, there exists the possibility of creating an ideal microenvironment that is suitable for cell engraftment and which could be used to reconstruct a new functional organ. In recent years, the whole organ decellularized scaffolds for the heart [11, 12], lungs [13], liver [14, 15], kidney [16], and pancreas [17] have been developed. In brief, the natural extracellular matrix and the release of growth factors contained in biological scaffold materials provide incomparable advantages, such as promoting cell adhesion and growth. Secondly, the natural vasculature of decellularized scaffolds permits continuous nutrition perfusion and enhances vascularization. Our previous studies have demonstrated that decellularized liver scaffolds could maintain the growth and function of isolated islets and islets-like clusters [18, 19]. Considering the growth factors secreted by specific ECM should be more suitable for the growth of original cells. We hypothesized the pancreatic structure should be a better platform for pancreatic *β*-like cells. We attempted to cultivate mouse GFP^+^-iPSCs derived pancreatic *β*-like cells using a decellularized rat pancreatic scaffold.

In the present study, we generated an ideal decellularized rat pancreatic scaffold. Mouse GFP^+^-iPSCs derived pancreatic *β*-like cells were obtained using appropriate growth factors via three steps. Following the cultivation of *β*-like cells in the decellularized scaffolds for five days, we evaluated the results via immunofluorescence and qRT-PCR. Our results indicated that mouse GFP^+^-iPSCs derived pancreatic *β*-like cells were well grown and functional. Moreover, the expression of insulin in this three-dimensional culture was higher than those results obtained using a two-dimensional culture. Taken together, our results suggest that pancreatic scaffolds can be used to optimize the generation of *β*-like cells derived from iPSCs and as such may represent a therapeutic means of curing diabetes mellitus.

## 2. Materials and Methods

### 2.1. Pancreas Harvest and Decellularization

All animal work was performed in accordance with the institutional guidelines and was approved by the Animal Ethics Committee of Nantong University. Sprague-Dawley (SD) rats weighing 250–300 g were used for the production of decellularized pancreatic scaffolds. All animals were kept on a 12 h light/dark cycle with free access to food and water. SD rats were anaesthetized by intraperitoneal injection of sodium pentobarbital intraperitoneally (1%, 1.2 mL/100 g) and anticoagulated by heparin 1000 U i.p. Following anesthetization, an abdominal incision was made. The celiac trunk and the communication branches between the spleen and intestine (stomach) were ligated and divided, and the splenic artery was inserted by an intravenous catheter (22 G) and fixed with 3-0 silk sutures. The cannula in the artery was connected to a peristaltic pump (Masterflex, Thermo Fisher, USA) to permit the flow of the perfusate. A total of 100 mL phosphate-buffered saline (PBS) was perfused at a speed of 2 mL min^−1^ to clear blood from the pancreas. Subsequently, the pancreas was perfused with 1% (w/v) Triton X-100 (Amresco)/0.1% ammonium hydroxide (Xilong Chemical Reagent Co., Ltd.) for 4 h at a speed of 2 mL min^−1^. Finally, the pancreas was perfused with PBS for 24 h to rinse cellular debris and maintain isotonicity. The decellularized pancreas scaffold was preserved in PBS containing 100 UmL^−1^ penicillin and 100 UmL^−1^ streptomycin at 4°C.

### 2.2. Decellularization Assessment

To examine the morphologic and histological features of the pancreatic scaffolds, tissues were randomly dissected from fresh pancreas and decellularized pancreatic scaffolds (*n* = 3) and fixed with 4% formaldehyde, dehydrated, and embedded in paraffin. For morphologic studies, tissue sections were deparaffinized and stained with hematoxylin and eosin (H&E) and Masson' trichrome. For immunohistochemistry, sample sections were blocked with 5% BSA for half an hour and 0.3% H_2_O_2_ to inhibit the endogenous peroxidase for 20 min. Subsequently, the sections were incubated with primary antibodies consisting of rabbit anti-collagen I 1 : 100 (Abcam), rabbit anti-collagen IV 1 : 100 (Abcam), rabbit anti-laminin 1 : 100 (Abcam), and rabbit anti-fibronectin 1 : 100 (Abcam) at 4°C overnight. The next day, the slides were incubated with secondary biotinylated goat anti-rabbit antibodies (Zsbio) and visualized using an Olympus microscope.

### 2.3. DNA and GAG Content Assay

For DNA quantification, the decellularized tissues and the fresh tissues (*n* = 5) were lyophilized and dissected into small pieces of approximately 20 mg. DNA was isolated according to the Dneasy Tissue kit protocol (Tiangen, China). The total amount of DNA was measured by ultraviolet spectrophotometer and the DNA content was calculated in the tissue. Quantification of GAG (glycosaminoglycan) was measured using the GAG assay kit (Hermes Criterion Biotechnology). Decellularized tissues and fresh tissues (*n* = 3) were dissected into small pieces weighing 10 mg and analyzed according to the manufacturer's instructions. Following ultraviolet spectrophotometer measurements, the GAG content was calculated in the tissues.

### 2.4. Scanning Electron Microscopy (SEM)

Decellularized and fresh pancreas were fixed in 2.5% glutaraldehyde in PBS overnight and subsequently washed three times, for 10 minutes each. The samples were fixed in the dark using 1% osmic acid for 2 h, followed by another three PBS washes for 10 min each. Subsequently, the samples were dehydrated in gradient series of alcohol for 15 min each. Subsequently, the samples were treated with isoamyl acetate and then sputter-coated with gold after critical point dried. Images were observed using scanning electron microscopy (HITACHI).

### 2.5. In Vivo Implantation of Decellularized Pancreas

Male C57BL/6 mice (*n* = 3), age 7-8 weeks, were anesthetized as mentioned above and the sterile scaffolds were sectioned into 5 × 5 × 2 mm^3^ sections to be implanted. The dorsal side was sterilized by iodophor and the surgical operation was performed under sterile conditions. A 1 cm incision was made in dorsal and a pocket similar to the implanted scaffolds was created to cover it. The incision was closed with 5-0 sutures and sterilized using iodophor for three days following the operation. At selected time points (3, 7, 14, 21, and 28 days), the sections were harvested and fixed in 4% paraformaldehyde for H&E staining.

### 2.6. Characterization of iPSCs

The mouse GFP^+^-iPSCs were kindly provided by Stem Cell Bank, Chinese Academy of Sciences [20]. Alkaline phosphatase staining and teratoma formation are usually implemented to identify the iPSCs. Teratoma formation is considered as the gold standard for confirming pluripotency of iPSCs [21]. For in vivo experiments, we injected 5 × 10^6^ mouse GFP^+^-iPSCs into the dorsal flanks of 5-week-old NOD/SCID mice (*n* = 6). Tumor formation was observed after three weeks and the tumors were resected on the fifth week. The samples were fixed with 4% formaldehyde, dehydrated, embedded in paraffin, and cut into 5 um thick sections. After being deparaffinized and stained with haematoxylin and eosin (H&E), alkaline phosphatase staining was implemented according to the instructions provided by the alkaline phosphatase detection kit (Millipore). Slides were visualized using an Olympus microscope.

### 2.7. In Vitro Differentiation of Mouse GFP^+^-iPSCs into Pancreatic *β*-Like Cells

Mouse GFP^+^-iPSCs were induced to differentiate into pancreatic *β*-like cells using a three-stages protocol described by Alipio et al. [9] and Schroeder et al. [22]. Stage one is as follows: GFP^+^-iPSCs were induced to embryoid bodies (EBs). GFP^+^-iPSCs were detached into single cell using 0.25% trypsin (sigma) and plated onto a 10 cm culture dish for 1 h to remove the feeder cells. The feeder-depleted cells were collected, centrifuged at 400*g* for 5 min, and resuspended into EBs medium containing knockout DMEM (Gibco), 15% FBS (sigma), 2 mM L-glutamine (Gibco), 1 × 10^−4^ M nonessential amino acids (Gibco), 1 × 10^−4^ M 2-mercaptoethanol (Sigma), and 1x penicillin-streptomycin (Gibco). The cells were suspended in EBs medium, 5000 cells per milliliter, transferred to ultra-low attachment plates (corning), and incubated for 3 days. Stage two is as follows: EBs were induced to multilineage progenitors. The EBs whose average size was 500 um were collected and transferred to 10 cm plates coated by 0.1% gelatin (sigma). Each plate contained 8–12 EBs and was incubated for another 9 days with EBs medium, which was replaced every 3 days. Stage three is as follows: EBs were induced to *β*-like cells. EBs medium was replaced by selective differentiation medium containing DMEM/F12 (corning), 15% FBS (sigma), 20 nM progesterone (Sigma), 100 *μ*M putrescine (Sigma), 1 *μ*g/mL laminin (Sigma), 10 mM nicotinamide (Sigma), 1x ITS (Gibco), B27 media supplement (Gibco), and 1x penicillin-streptomycin (Gibco). On the sixth day, the cells were trypsinized and transferred into T25 tissue culture flask for 14 days with medium changed every 3 days.

### 2.8. Characterization of Pancreatic *β*-Like Cells

The mouse GFP^+^-iPSCs derived *β*-like cells were characterized by immunofluorescence (IF) and qRT-PCR. For IF, cells were fixed by 4% paraformaldehyde for 20 min, permeabilized by 0.1% Triton X-100 for 10 min, and blocked with 5% BSA for 30 min. After that, the cells were incubated with primary antibodies overnight at 4°C including rabbit anti-insulin 1 : 100 (Abcam), rabbit anti-C-peptide 1 : 100 (Abcam), and rabbit anti-Glucagon 1 : 100 (Abcam). The next day, the cells were washed three times in PBS and incubated with secondary antibodies including Alexa Flour 594-conjugated goat anti-rabbit 1 : 500 for insulin, c-peptide, and glucagon at room temperature for 1 hour. Subsequently, the cells were stained with Hoechst (sigma) for 15 min followed by 3 changes of PBS changes. The cells were visualized using an Olympus fluorescence microscope. For qRT-PCR, TRIzol reagent (Life Technologies) was used to extract total RNA from *β*-like cells, according to the manufacturer's instruction. RNA was reverse-transcribed into cDNA using RevertAid™ First Strand cDNA Synthesis Kit (Fermentas). Gene expression levels of insulin, glucagon, PDX-1, islet-1, Nkx6.1, and HNF6 were analyzed by qRT-PCR. The reaction mixtures included 10 *μ*L SYBR Green Master (Roche), 4 *μ*L Template DNA, 1 *μ*L forward primer, 1 *μ*L reverse primer, and 4 *μ*L DEPC water. PCR primer sequences for the specific pancreatic markers are shown in Table 1. The 7500 Real-Time PCR System (Applied Biosystems) was used for cDNA amplification. The amplification protocol included 5 min at 94°C for initial denaturation, followed by 40 cycles of 30 s at 94°C for denaturation, 30 s at 60°C for annealing, and final extension at 72°C for 30 s. To prove whether the differentiated pancreatic beta-like cells were teratogenic after differentiation, teratoma formation was taken as mentioned above.

### 2.9. Insulin Release Assays

Stage-three cells were trypsinized and transferred into new Petri dishes for 12 h. After being preincubated in glucose-free Krebs-Ringer bicarbonate buffer (KRB) for 90 min, the cells were incubated with KRB containing 0, 5, 15, 30, and 45 mM glucose for 90 min. The supernatant was collected and frozen at −80°C to be detected. Insulin assay was performed by ultrasensitive mouse insulin assay kit (Mercodia) according to the manufacturer's instruction.

### 2.10. Mouse GFP^+^-iPSCs Derived *β*-Like Cells Seeding and Dynamic Culturing

Before seeding *β*-like cells, the sterile scaffold was perfused with a medium consisting of DMEM/F12 with 15% FBS through the circulation perfusion device for 1 hour. *β*-like cells were plated into scaffolds using two methods including vascular perfusion and multipositional parenchymal injection as previously described. *β*-like cells (3 × 10^7^) were trypsinized and suspended in 1 mL of medium. Approximately 0.4 mL of medium was injected into the decellularized pancreatic scaffold through the splenic artery and 0.6 mL medium through the multipositional parenchymal injection. The scaffold was allowed to incubate for 30 min to facilitate cell adherence. The medium was subsequently seeped out from the scaffold and the remaining cells were resuspended in 1 mL of medium to reperfuse the scaffold. The scaffold was allowed to incubate for 2 h prior to being linked to the circulation perfusion device. Finally, the scaffold was linked to the device through splenic artery to allow perfusion feeding of media at the speed of 1.2 mL/min. After five days of culture at 37°C, the seeded scaffolds were analyzed using HE staining, immunofluorescence, and qRT-PCR as previously described.

### 2.11. Statistical Analysis

Statistical analysis was performed using SPSS 19 software. Statistical differences were identified using a Kruskal-Wallis test when *p* value of less than 0.05 was obtained.

## 3. Results

### 3.1. Perfusion Decellularization of Rat Pancreas

**Figure 1:**
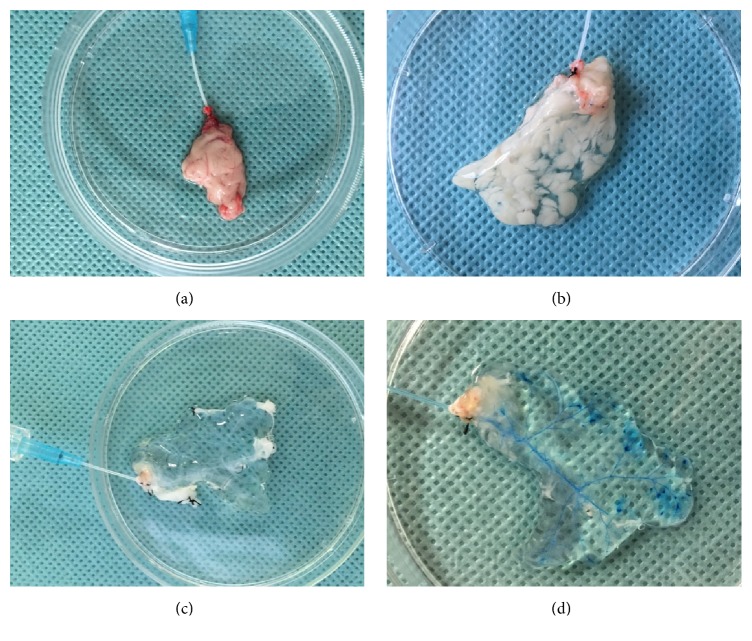
Pancreas harvest and decellularization. (a) The pancreas was separated from adjacent tissue. (b) Following perfusion with PBS, the pancreas became semitransparent. (c) Following perfusion with 1% Triton X-100/0.1% ammonium hydroxide, the pancreas quickly became transparent and maintained the original shape. (d) Vasography clearly showed the vascular system.

**Figure 2:**
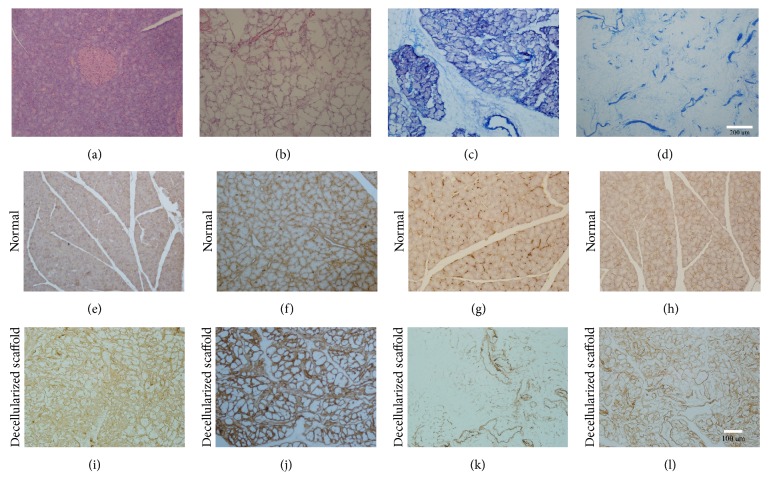
Analyzation of ECM components. (a, b) H&E staining of the normal pancreas and decellularized pancreatic scaffold. (c, d) Masson's trichrome of the normal pancreas and decellularized pancreatic scaffold. Immunohistochemical analysis of the normal pancreas and decellularized pancreatic scaffold: (e, i) collagen I, (f, j) collagen IV, (g, k) fibronectin, and (h, l) laminin.

**Figure 3:**
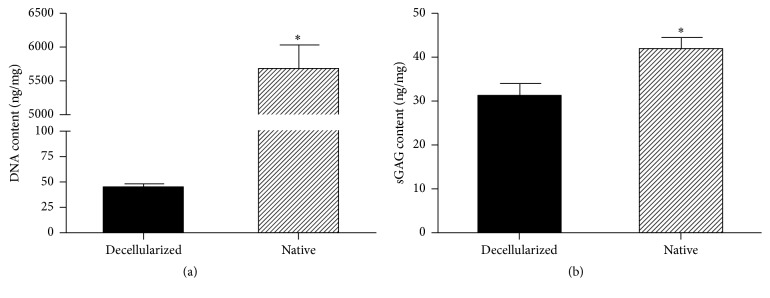
DNA and GAG content assay. (a) Quantitative DNA analysis demonstrated that the DNA content of the decellularized pancreas scaffolds was 43.96 ± 4.07 ng/mg dry weight in contrast to 5666 ± 360.9 ng/mg for the native pancreas (^*∗*^*p* < 0.05). (b) The GAG content in the decellularized pancreatic scaffolds was 30.9 ± 2.93 ng/mg wet weight compared to 41.9 ± 2.74 ng/mg in the normal pancreas (^*∗*^*p* < 0.05).

SD rat pancreases were decellularized using 10% Triton X-100/0.1% ammonium hydroxide solution. A gradual change of color was observed during the decellularization process. The blood was first washed out and the pancreas turned semitransparent after 15 minutes of perfusion with PBS (Figure 1(b)). Following perfusion with 1% Triton X-100/0.1% ammonium hydroxide (about 3 h), the pancreas quickly became transparent (Figure 1(c)) and the anatomical structure of decellularized pancreas was well retained and visualized according to the vasography and anatomic microscope (Figure 1(d)). H&E staining showed no residual cells following decellularization, as compared to the native pancreas (Figures 2(a) and 2(b)). The result of Masson's trichrome staining also confirmed that most of the collagen fiber components and vascular structure were well retained (Figures 2(c) and 2(d)). Immunohistochemical analysis of ECM in the native pancreas and scaffold demonstrated that collagen I (Figures 2(e) and 2(i)), collagen IV (Figures 2(f) and 2(j)), fibronectin (Figures 2(g) and 2(k)), and laminin (Figures 2(h) and 2(l)) were preserved following complete decellularization. Quantitative DNA analysis demonstrated that the DNA content of decellularized pancreas scaffolds was 43.96 ± 4.07 ng/mg dry weight in contrast to 5666 ± 360.9 ng/mg for the native pancreas (Figure 3(a)) (^*∗*^*p* < 0.05). The GAG content in the decellularized pancreatic scaffolds was 30.9 ± 2.93 ng/mg wet weight compared to 41.9 ± 2.74 ng/mg wet weight in normal pancreas. These results confirmed that about 70% of GAG was preserved in the decellularized pancreatic scaffold after the decellularization (Figure 3(b)) (^*∗*^*p* < 0.05).

### 3.2. SEM Examination

**Figure 4:**
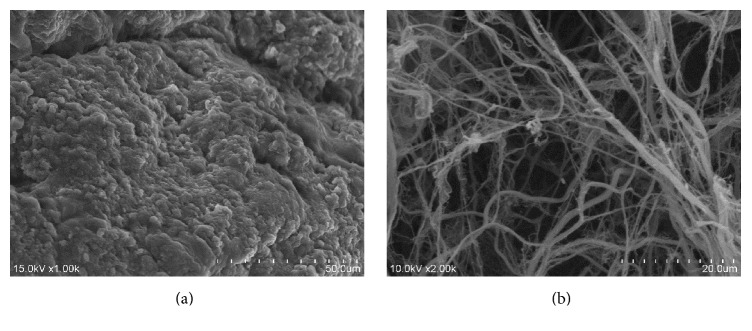
Scanning electron microscopy. (a, b) SEM showed the microstructure of the native and decellularized pancreatic scaffolds. No residual cells were retained and the integrity of microstructure were largely preserved.

The microstructure of the native and decellularized scaffolds was evaluated by SEM. No residual cells were retained and the integrity of three-dimensional ECM microstructure of the decellularized pancreatic scaffolds was largely preserved (Figures 4(a) and 4(b)). In general, the decellularized rat pancreas scaffolds possessed a microstructure similar to the native pancreas.

### 3.3. Biocompatibility Assay

**Figure 5:**
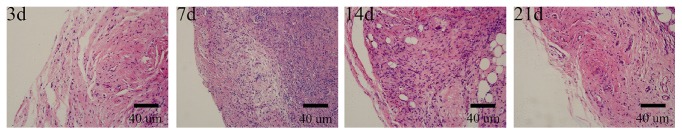
Biocompatibility assay. Inflammatory cells began to infiltrate into the scaffold on day 3, reaching a peak on day 7. The inflammatory response subsequently began to reduce and there were few inflammatory cells on day 21. The scaffolds retained their appearance and texture.

To evaluate the in vivo biocompatibility of the decellularized rat pancreatic scaffolds, the implanted scaffolds were removed on the following schedule after surgery (days 3, 7, 14, and 21). The scaffolds retained their appearance and texture. H&E staining revealed mononuclear cells that began to permeate through the scaffold by day 3 and reached the peak on day 7. The number of inflammatory cells diminished until there were a few remaining by day 21 (Figure 5). Throughout the entire observation period, few macrophagocytes or other pathological signs were observed, suggesting that the decellularized pancreas scaffolds were biocompatible.

### 3.4. Identification of iPSCs and Pancreatic *β*-Like Cells

**Figure 6:**
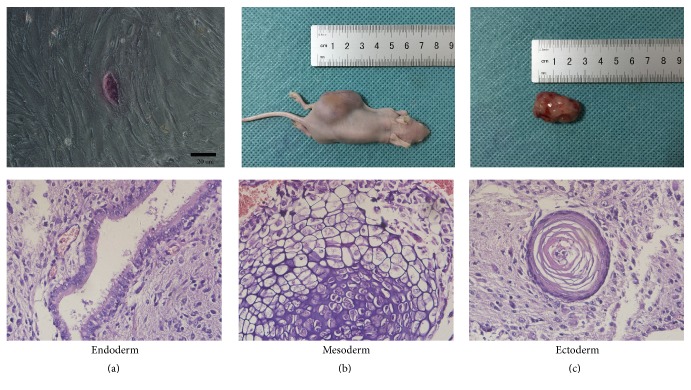
Identification of iPSCs. (a) The GFP^+^-iPSCs were purple which showed positive alkaline phosphatase staining. (b, c) Five weeks following injection, a 2.5 × 1.5 × 1.5 cm^3^ size tumor formed. Using hematoxylin and eosin staining, tumor tissue was noted to be derived from all three embryonic layers, including glandular epithelium (endoderm), cartilage (mesoderm), and cornified epithelium (ectoderm).

For alkaline phosphatase staining, the GFP^+^-iPSCs were purple which showed positive alkaline phosphatase staining (Figure 6(a)). For teratoma formation, 5 × 10^6^ mouse GFP^+^-iPSCs were injected into the dorsal flanks of 5-week-old NOD/SCID mice. Five weeks following injection, a tumor of 2.5 × 1.5 × 1.5 cm^3^ in size was formed and resected (Figures 6(b) and 6(c)). Using hematoxylin and eosin staining, we determined that tumor tissue was derived from all three germinal layers, including glandular epithelium (endoderm), cartilage epithelium (mesoderm), and cornified epithelium (ectoderm). When it came to the pancreatic *β*-like cells, there was no teratoma formation in all NOD/SCID mice.

### 3.5. Selective Differentiation of Mouse GFP^+^-iPSCs into Pancreatic *β*-Like Cells

**Figure 7:**
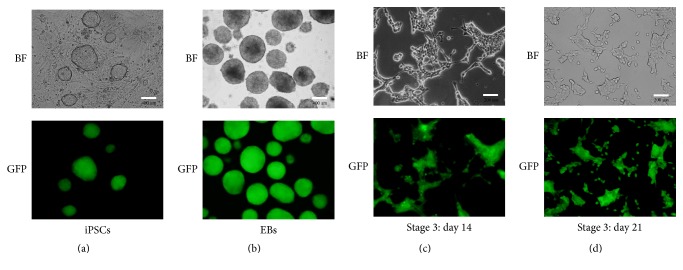
Selective differentiation of iPS cells into insulin-producing pancreatic *β*-like cells. Morphologies of mouse GFP^+^-iPSCs differentiated into *β*-like cells: (a) undifferentiated GFP^+^-iPSCs. (b) In the first stage, GFP^+^-iPSCs resuspended on ultra-low attachment plates for three days forming EBs. (c) The third stage on day 14: differentiated cells grew together closely. (d) The third stage on day 21: cells grew well and gathered liked islets.

**Figure 8:**
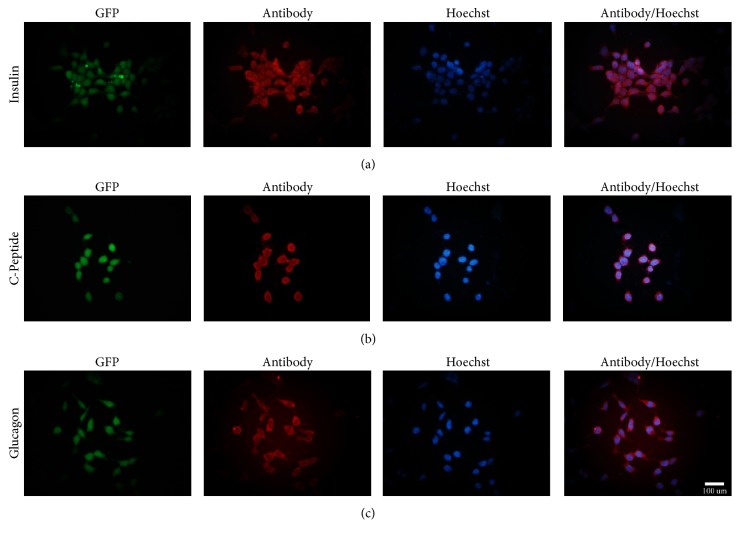
Immunofluorescent of GFP^+^-iPSCs derived *β*-like cells. (a, b, c) The GFP^+^-iPSCs derived *β*-like cells were positive for insulin, C-peptide, and glucagon.

**Figure 9:**
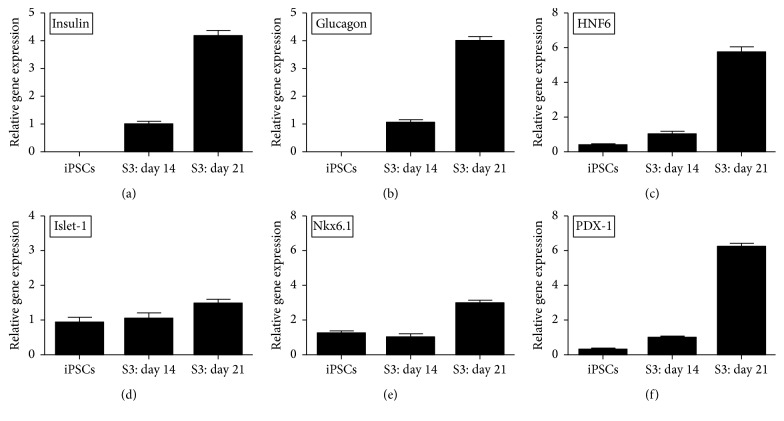
qRT-PCR by the *β*-like cells in vitro. (a–f) qRT-PCR demonstrated elevated expression of insulin, glucagon, HNF6, islet-1, Nkx6.1, and PDX-1 on day 21 compared with day 14 and undifferentiated iPSCs.

**Figure 10:**
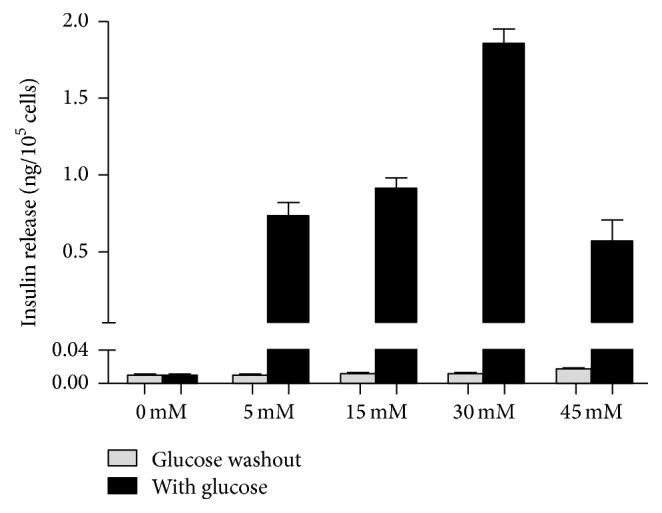
Insulin release assays. Insulin secretion was 0.724 ± 0.098 ng/10^5^ cells, 0.902 ± 0.078 ng/10^5^ cells, 1.844 ± 0.102 ng/10^5^ cells, and 0.558 ± 0.150 ng/10^5^ cells at a glucose concentration of 5 mM, 15 mM, 30 mM, and 45 mM. By contrast, no insulin was produced when glucose was washed out.

Mouse GFP^+^-iPSCs were differentiated into *β*-like cells in three stages. At stage 1, GFP^+^-iPSCs were detached into single cells and resuspended in EBs medium in ultra-low attachment plates for three days, where they formed EBs (Figure 7(b)). At stage 2, the EBs were transferred to an adherent culture and differentiated into multilineage progenitors for 9 days. During this time, cells migrated from EBs and grew by adherence. At stage 3, which lasted 21 days, the cells were trypsinized and resuspended in fresh selective medium. Numerous clusters began to form on day 14 (Figure 7(c)), and on day 21, large clusters were evident (Figure 7(d)). To evaluate the efficiency of differentiation, immunofluorescent staining and qRT-PCR were performed. The cells were positive for insulin, C-peptide, and glucagon by immunofluorescence (Figure 8). qRT-PCR confirmed the immunofluorescence findings, indicating higher expression of the previously mentioned markers on day 21 as compared to day 14 controls and GFP^+^-iPSCs (Figures 9(a) and 9(b)). Hormones expressed by pancreatic endocrine cell included *α* and *β*, suggesting that the differentiated cells had progressed into mature *β*-like cells. Also, some reliable markers in the development of the pancreatic *β* cells including PDX-1, islet-1, Nkx6.1, and HNF6 were expressed higher on day 21 compared to day 14 and the undifferentiated iPSCs (Figures 9(c)–9(f)). To decide whether the differentiated cells were glucose responsive, the *β* cells were exposed to a glucose gradient (0 mM, 5 mM, 15 mM, 30 mM, and 45 mM) and insulin release was measured by ELISA assay. At a glucose concentration of 0 mM, insulin secretion was hardly detected. However, at the 5 mM, 15 mM, 30 mM, and 45 mM glucose concentration, insulin was detected at 0.724 ± 0.098 ng/10^5^, 0.902 ± 0.078 ng/10^5^, 1.844 ± 0.102 ng/10^5^, and 0.558 ± 0.150 ng/10^5^ cells, respectively (Figure 10).

### 3.6. GFP^+^-iPSCs Derived *β*-Like Cells in Decellularized Pancreatic Scaffolds

**Figure 11:**
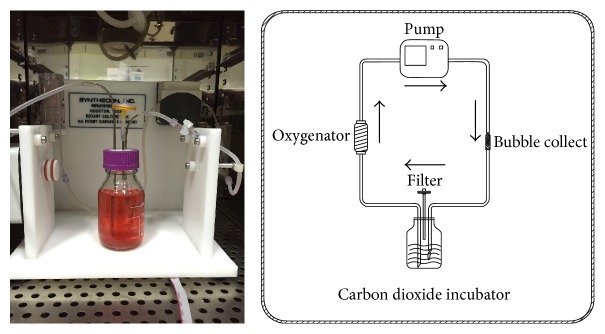
*β*-like cells were planted into scaffolds. The perfusion device consisted of peristaltic pump, oxygenator, bubble collect, culture bottle, and convey tubes. The GFP^+^-iPSCs derived *β*-like cells in the scaffold were cultured for 5 days.

**Figure 12:**
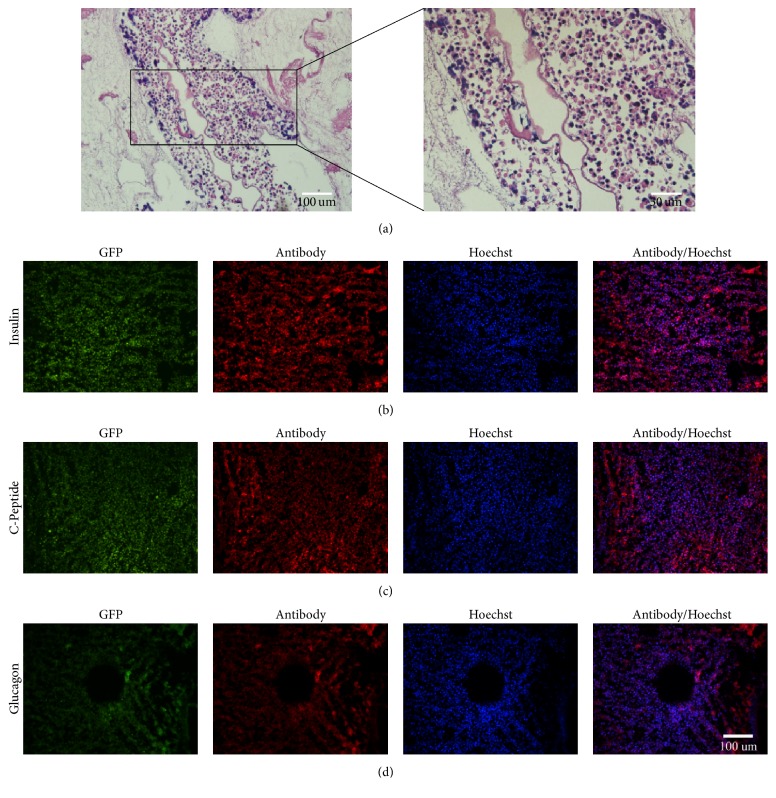
GFP^+^-iPSCs cell-derived *β*-like cells in decellularized pancreatic scaffolds. (a) HE staining of recellularization of decellularized pancreatic scaffolds showed GFP^+^-iPS derived *β*-like cells were settled around the vascular lumen. (b, c, d) Immunofluorescence demonstrated positivity for insulin, C-peptide, and glucagon thus demonstrating that engrafted cells could survive and function in the scaffolds.

**Figure 13:**
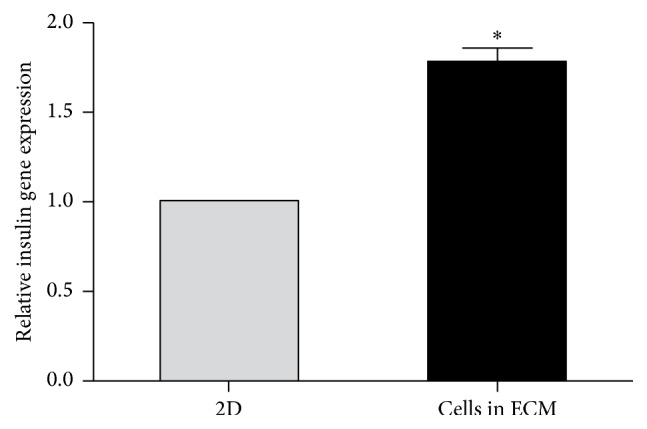
Relative insulin gene expression in 2D compared to 3D. Relative insulin gene expression in 3D cultured in the pancreatic scaffold was significantly higher than that of the 2D (^*∗*^*p* < 0.05).

To evaluate GFP^+^-iPSCs derived *β*-like cells in decellularized rat pancreatic scaffolds, *β*-like cells were plated into scaffolds using two methods: vascular perfusion and multipositional parenchymal injection. Approximately 3 × 10^7^ cells were plated into the scaffolds with a total engraftment rate of 83.6 ± 4.8%. Following stewing for 2 h, the scaffold was linked to the dynamic perfusion device through splenic artery with culture media which was changed daily (Figure 11). After five days of perfusion culture, the scaffold was harvested and analyzed by H&E staining, IHC, and qRT-PCR. H&E staining revealed that *β*-like cells engrafted the parenchymal region (Figure 12(a)) and IHC demonstrated that engrafted cells maintained insulin, C-peptide, and glucagon expression (Figures 12(b), 12(c), and 12(d)). Moreover, insulin expression in the perfusion culture was nearly 2-fold higher than those levels obtained by traditional culturing (^*∗*^*p* < 0.05) (Figure 13). Taken together, these results demonstrate that the decellularized pancreas is cytocompatible and maintainable of function.

## 4. Discussion

Our research focused on combining iPSCs derived pancreatic *β*-like cells with whole organ acellular pancreatic scaffold methods. By simulating the in vivo microenvironment, we confirmed the differentiation and functionality of iPSCs into *β*-like cells. These results provide a novel means of exploring the function and efficacy of generating iPSCs derived pancreatic *β*-like cells in vitro as in vivo and, importantly, may provide a means of curing diabetes mellitus via recellularized pancreatic scaffolds.

Currently, stem cells including pancreatic islet stem cells, embryonic stem cells, mesenchymal stem cells, and induced pluripotent stem cells [23] have been suggested as a means to regenerate the pancreas. This approach has been deemed the most promising way of solving overcoming limitations associated with diabetes-related transplantation. iPSCs, differentiated with multidirectional potential, can resemble ES cells, via reprogramming of somatic cells [24]. This revolutionary discovery has had a broad impact on pathology research [25], drug screening, cell transplantation therapy [26], tissue engineering, and regenerative medicine [27]. iPSCs can be derived from host somatic cells, are easy to obtain, can expand rapidly, and, most importantly, have lower immunogenicity as compared to transplants using donor-derived cells or tissues. In addition, iPSCs avoid ethical implications which have clouded the use of embryonic stem cells [28]. iPSCs are usually identified utilizing alkaline phosphatase staining, immunostaining of makers of stem cells, EB formation, teratoma formation, Karyotype analysis, and so on. Teratoma formation is considered as the gold standard for confirming pluripotency of iPSCs. Nude mouse tumorigenicity assay proved the multipotent capacity of the iPSCs [29, 30]. For the past few years, iPSCs have been successfully differentiated into neurons [31], cardiac muscle cells [32], and hematopoietic cells [33] among other cell types. While the current most effective strategy to cure diabetes is islet or *β* cell transplantation, the shortage of donor, low survival rate of the grafted cells, and immune rejection following transplantation limit the success of this approach. As such, the use of reprogrammed *β* cells derived from autologous stem cells has tremendous potential [34] to solve the transplant-related shortage of the donor and also bypass the immune rejection associated with transplantation. In the present study, we adopted the three-step procedure described by Alipio et al. [9] to induce GFP^+^-iPSCs into pancreatic *β*-like cells. GFP^+^-iPSCs were resuspended on ultra-low attachment plates for three days to form EBs. Compared to other methodologies, this approach was easy to operate and generated more EBs. EBs were subsequently transferred onto an adherent culture for nine days where EBs differentiated into multilineage progenitors. Through the use of selective medium over the course of a 21-day period, the cells developed into pancreatic *β* cells. Immunofluorescence and qRT-PCR independently confirmed the presence of insulin, C-peptide, and glucagon which reached its peak on day 33. Also, some reliable markers in the development of the pancreatic *β* cells were expressed higher on day 21 compared to day 14 and the undifferentiated iPSCs. PDX-1 plays a crucial role in beta-cell differentiation and transactivates the insulin gene [35]. During the pancreas development, PDX-1 is expressed in precursor cells but becomes restricted to *β*-cells in mature pancreas. Islet-1 is an important transcription factor expressed in pancreatic islet cells [36]. It is important for the survival and differentiation of pancreatic endocrine progenitors. Lack of islet-1 will affect insulin secretion and islet transcriptome. Nkx6.1 is essential for maintaining the functional and molecular traits of mature beta cells [37]. HNF6 directly activates the proendocrine transcription factor and is significant for endocrine differentiation [38]. Those improving expressions of crucial gene in the development of pancreas indicated that the differentiated model is mature. Previous groups have used different factors to induce differentiation including fibroblast growth factor, epidermal growth factor, retinoic acid, and activin A, but these may induce neuronal (i.e., nerve cell) differentiation [39]. Our experiment approach conversely does not add these factors and as such avoids these risks. The insulin secretion was 1.844 ± 0.102 ng/10^5^ cells when exposed to the glucose concentration of 30 mM. It needs about 3.1–3.4 × 10^5^ cells to produce a physiological range of insulin content since the mouse blood volume is about 1.5 mL and its physiological value of insulin is about 4 *μ*g/L. While the approach is promising, more research is needed to further characterize the in vitro differentiation process. As the seeded cells for tissue engineering, optimizing the differentiated cells is of great importance.

Currently, a number of biomaterial scaffolds are being used to evaluate cell function in vitro including hydrogels, chitosan, silk fibroin, collagen type I gel sandwich culture [40], and 3D matrices. In spite of the advantages of these models, these systems cannot evaluate the long-term cell biology, physiology, and chronic effects of toxicity. In recent years, whole organ decellularized scaffolds have been proposed as a novel means of ameliorating scaffold-based analysis. Decellularized scaffolds not only provide space for cell growth but also promote cell migration, differentiation, and metabolism through interactions with the cell matrix. Ion detergent SDS and nonionic detergent Triton-100 are frequently used to produce decellularized scaffolds. As a powerful detergent, SDS can quickly remove cells but at the same time may damage the ECM to a large extent. Triton-100 acts as a more mild detergent and thus better preserves the ECM making this approach more suitable for the development of scaffolds from the liver, pancreas, spleen, kidney, and lung. We adopted 1% Triton X-100/0.1% ammonium hydroxide [41] as the perfusate and controlled the perfuse flow rate at a speed of less than 2 mL/min. After 15 min of washing with PBS, blood was washed out and the pancreas became white. Following an additional three hours of perfusion using 1% Triton X-100/0.1% ammonium hydroxide, the pancreas was completely transparent. Increasing the efficiency could reduce the loss of the ECM components and improve the quality of scaffolds. To verify our results, H&E analysis was performed and revealed no basic cell nucleus. The dsDNA content was also just 43.96 ± 4.07 ng/mg. Residual cell components, such as protein, will trigger a host immune response [42]; therefore, less vestigial cells will relieve the host immune rejection and improve the biocompatibility. In our xenotransplantation experiment, we confirmed the decellularized pancreatic scaffolds as biocompatible. The specimens were examined by HE staining, for up to 21 days after operation. We observed early inflammatory infiltrates on day 3, reaching their peak on day 7, after which point the response began to diminish. Notably, the host response was significantly reduced by day 21. At the same time, the pancreatic scaffold structures were well preserved, confirming that the method we adopted was effective and safe. Protein and polysaccharide in the ECM are essential to reconstruct and restore the organization. Immunofluorescence demonstrated that collagen I, collagen IV, fibronectin, and laminin were well preserved. The GAG content in the decellularized pancreas scaffolds was 70%, compared to the normal pancreas.

Our preliminary work has revealed that 3D decellularized scaffolds of islets performed better than the 2D cultures [18, 19]. Considering specific ECM should be more suitable for the growth of original cells [43, 44]. We hypothesized that decellularized pancreatic scaffolds should represent a superior platform to support *β*-like cells growth and may provide an alternative source of donor organs. After GFP^+^-iPSCs derived pancreatic *β*-like cells were transplanted into scaffolds, the scaffolds were connected to our circle culture system via splenic artery. Continuously nutrient solution and oxygen were perfused into the scaffolds to support cell growth as in vivo. IF confirmed the presence of insulin, C-peptide, and glucagon from our GFP^+^-iPSCs derived *β*-like cells. Moreover, there was a significant increase in the expression of insulin as compared to traditional 2D culture. Taken together, our results allow us to conclude that the 3D decellularized bioscaffold is a promising material to promote cell growth in vivo.

In our study, we evaluated the idea that GFP^+^-iPSCs derived pancreatic *β*-like cells were well grown and functional in the decellularized pancreatic scaffolds. This makes it possible to evaluate iPSCs derived cells in vitro as in vivo, enhancing our abilities to apply these techniques to various modalities of research such as disease pathology, drug screening, and cell transplantation therapy. The application of stem cell and decellularized scaffolds represents an extremely promising step forward in the search for a cure for diabetes. However, cultivating iPSCs derived pancreatic *β*-like cells using decellularized pancreatic scaffolds will be the preliminary work and there is still a long road ahead before the applications of this methodology in clinical practice. Namely, if iPSCs derived pancreatic *β*-like cells are to be differentiated and cultured in the scaffolds, it would be significant to explore what growth factors are left after decellularization, since it may affect directly the survival and differentiation of pancreatic *β*-like cells. Secondly, our research demonstrated that transplanted cells did not distribute uniformly, although vascular perfusion and multipositional parenchymal injection were taken simultaneously. Also revascularization remains a major bottleneck towards biological engineering, and while we have promising preliminary data towards addressing this issue, more works still need to be performed in future studies [45].

## 5. Conclusion

Our research reveals that the iPSCs will play a crucial role in the regenerative medicine and tissue engineering. On account of deriving from host somatic cells, it will solve those problems such as lack of cell sources and immunological rejection. With selective differentiation medium, it is feasible to obtain the pancreatic *β*-like cells. Since it is easy and effective to generate decellularized pancreatic scaffolds nowadays, utilizing the whole organ acellular pancreatic scaffolds will help us explore the cell-ECM interactions in vitro as in vivo, making a novel method to optimize the *β*-like cells. In the further, there is more needed to be done such as analyzing mechanisms of differentiation in the decellularized scaffolds. Also, exploring the methods to recellularize the decellularized pancreatic scaffolds with *β*-like cells will be a breakthrough in the treatment of diabetes mellitus.

## Figures and Tables

**Table 1:** PCR primer sequences for specific pancreatic markers.

Gene	GenBank accession number	Primer
GAPDH	NM_000072.6	
Forward primer		AAGAAGGTGGTGAAGCAGG
Reverse primer		GAAGGTGGAAGAGTGGGAGT
Insulin	NM_000085.6	
Forward primer		AGTTGAGTTGGGCAGAATAGG
Reverse primer		TCCAAAGGGCACCGTAT
Glucagon	NM_000068.7	
Forward primer		CCAGCGACTACAGCAAATACC
Reverse primer		GAGAAGGAGCCATCAGCGT
HNF6	NM_000075.6	
Forward primer		CGTTACAGCATCCCACAG
Reverse primer		AGCCACTTCCACATCCTC
Nkx6.1	NM_144955.2	
Forward primer		GAAAACACACCAGACCCA
Reverse primer		GGAACCAGACCTTGACCT
Islet-1	NM_021459.4	
Forward primer		CACCTTGCGGACCTGCTAT
Reverse primer		AGGGCGGCTGGTAACTTTG
Pdx-1	NM_008814.3	
Forward primer		CGGAACCCGAGGAAAACA
Reverse primer		CGAGGTCACCGCACAATCT
